# PAS or Not PAS? The Sonographic Assessment of Placenta Accreta Spectrum Disorders and the Clinical Validation of a New Diagnostic and Prognostic Scoring System

**DOI:** 10.3390/jimaging10120315

**Published:** 2024-12-10

**Authors:** Antonella Vimercati, Arianna Galante, Margherita Fanelli, Francesca Cirignaco, Amerigo Vitagliano, Pierpaolo Nicolì, Andrea Tinelli, Antonio Malvasi, Miriam Dellino, Gianluca Raffaello Damiani, Barbara Crescenza, Giorgio Maria Baldini, Ettore Cicinelli, Marco Cerbone

**Affiliations:** 1 Obstetrics and Gynaecology Unit, Department of Interdisciplinary Medicine (DIM), University of Bari, 70124 Bari, Italybarbaracrescenza@gmail.com (B.C.);; 2 Chair of Medical Statistic, Department of Interdisciplinary Medicine (DIM), University “Aldo Moro” of Bari, 70124 Bari, Italy; 3 Department of Obstetrics and Gynecology and CERICSAL (CEntro di RIcerca Clinico SALentino), Veris Delli Ponti Hospital, 73020 Scorrano, Italy

**Keywords:** placenta accreta spectrum, PAS, placenta previa, PAS score system, hysterectomy, riddled cervix sign, retroplacental myometrial thinning, vascular lacunae, retroplacental vascularization

## Abstract

This study aimed to evaluate our center’s experience in diagnosing and managing placenta accreta spectrum (PAS) in a high-risk population, focusing on prenatal ultrasound features associated with PAS severity and maternal outcomes. We conducted a retrospective analysis of 102 high-risk patients with confirmed placenta previa who delivered at our center between 2018 and 2023. Patients underwent transabdominal and transvaginal ultrasound scans, assessing typical sonographic features. Binary and multivariate logistic regression analyses were performed to identify sonographic markers predictive of PAS and relative complications. Key ultrasound features—retroplacental myometrial thinning (<1 mm), vascular lacunae, and retroplacental vascularization—were significantly associated with PAS and a higher risk of surgical complications. An exceedingly rare sign, the “riddled cervix” sign, was observed in only three patients with extensive cervical or parametrial involvement. Those patients had the worst surgical outcomes. This study highlights the utility of specific ultrasound features in stratifying PAS risk and guiding clinical and surgical management in high-risk pregnancies. The findings support integrating these markers into prenatal diagnostic protocols to improve patient outcomes and inform surgical planning.

## 1. Introduction

Placenta accreta spectrum (PAS) encompasses a range of placental disorders characterized by the abnormal adherence or invasion of trophoblastic tissue into the myometrium. [1,2]. These disorders include placenta accreta, placenta increta, and placenta percreta, each representing a different degree of invasion of the trophoblast into the myometrium [3]. The current evidence considers cesarean scar pregnancy (CSP) and PAS to be different aspects of the same condition, showing higher risks with advancing gestational age [4,5]. PAS disorders pose significant risks to both maternal and fetal health, varying from preterm birth to uterine rupture, severe postpartum hemorrhage, and, in the most severe cases, hysterectomy, with high rates of maternal mortality and morbidity [6,7]. Placenta previa and a prior cesarean delivery stand as the primary risk factors for PAS, with the occurrence of PAS rising dramatically with each additional cesarean procedure [8]. As the rate of cesarean deliveries continues to grow, the prevalence of abnormal placentation is on a steady rise. Estimates suggest that the incidence of PAS is increasing by 30% every 10 years [7,8]. Other known risk factors for PAS include procedures that may damage the endometrium, including uterine curettage, myomectomy, hysteroscopic lysis of intrauterine adhesions, endometrial ablation, uterine-artery embolization, or the manual removal of the placenta [3,9]. Risk factors concerning the medical history of the patients include abnormally adherent placenta in a previous pregnancy, advanced maternal age, increased parity, and in vitro fertilization [10,11]. Accurate diagnosis of PAS is challenging, particularly in less severe cases [12,13,14]. Today, an ultrasound scan is the preferred screening tool for PAS, but a lack of consensus on the diagnostic criteria and interobserver variability in interpreting ultrasound features further complicate management, especially in high-risk populations such as those with placenta previa [15,16,17]. The International Federation of Gynaecology and Obstetrics (FIGO) recently introduced a novel classification system for the clinical diagnosis of PAS [2]. This new classification is utilized when PAS is identified either during vaginal delivery or during cesarean section. However, it remains uncertain whether it correlates with significant hemorrhage in cases of placenta previa complicated by PAS [18,19,20]. Currently, prenatal diagnosis relies on the subjective interpretation of “typical” sonographic findings or signs [5]. Magnetic resonance imaging (MRI), although widely employed, has yet to clearly demonstrate a significant improvement in the diagnosis of PAS disorders [21,22]. Currently, the interobserver variability in the interpretation of ultrasound findings related to placental invasion is significant, and regarding the diagnostic criteria, there is a lack of consensus among scientific societies, so there is an urgent need for a standardized approach to diagnosing PAS [23,24,25]. While various scoring systems and diagnostic criteria have been proposed, such as the “Placenta Accreta Index”, “ultrasound staging system for PAS”, and the “two-criteria system”, their clinical utility is hindered by limitations, such as sample selection bias and inconsistent scoring indicators [26,27,28,29]. Additionally, the diagnostic performance of these systems varies across different degrees of placental invasion in PAS, which further complicates their practical application.

## 2. Study Aim

Despite advances in imaging, an accurate diagnosis of PAS remains a clinical challenge, necessitating the development of more robust diagnostic criteria. In this context, our study aims to establish a correlation between the presence of the main clinical risk factors, sonographic features, and intraoperative findings of PAS, as well as intraoperative complications. This entailed investigating whether the various clinical and imaging parameters were capable of predicting PAS in surgical specimens and establishing which ones performed best. Additionally, this study aims to assess whether these parameters correlate with the occurrence and severity of intraoperative complications encountered during the surgical management of PAS. By elucidating these relationships, the study aims to enhance the clinical utility of the risk scoring system for PAS, aiding in more precise risk stratification and informing surgical decision-making.

## 3. Methods

### 3.1. Patient Enrollment and Data Collection

In this retrospective, single-center study, we included a cohort of consecutive patients with a very high risk for placenta accreta due to the invariable presence of placenta previa, who were undergoing routine evaluation and delivery in the Gynecology and Obstetrics Unit of University of Bari “Aldo Moro” (South Italy). The study period was between January 2018 and December 2023; every patient underwent multiple routine prenatal ultrasound scans to evaluate placental invasion. Relevant data concerning the medical history of the patients were recorded in the clinical charts (any previous cesarean section, uterine myomectomy, uterine curettage, in vitro fertilization, maternal age, gestational age at delivery, intraoperative blood loss, and histopathologic details). An anonymous database was then filled with these data.

### 3.2. Inclusion and Exclusion Criteria

The inclusion criteria were women diagnosed with singleton pregnancy with placenta previa who underwent obstetric checks at our department. We excluded patients with multiple pregnancies and major systemic and obstetric pathologies such as complicated diabetes, severe pre-eclampsia, or coagulopathies. Cases with incomplete evaluations and those lost to follow-up were excluded from the study.

### 3.3. Sonographic Technique and Features

Women were evaluated by transabdominal and transvaginal ultrasound (Voluson E10, GE Medical Systems©, Chicago, IL, USA) using a system equipped with a 4 to 8 MHz transducer. Patients were blindly evaluated by an expert sonographer and a senior resident, and their assessments were reported and subsequently discussed to set an agreement. The sonographic features that we evaluated were the placental location (anterior/posterior), presence/absence of exophytic mass, presence/absence of at least three placental lacunae, presence/absence of the retroplacental space, presence/absence of bridging vessels, thickness of the retroplacental myometrium (<1 mm), bladder line interruption, retroplacental myometrial blood flow, presence/absence of intracervical lakes, and cervical length. We attributed a numeric grade between 0 and 1 to each sonographic feature, modifying the existing scoring system from Zhang et al. [29] (Table 1).

We applied the sonographic techniques outlined by MWF Rac et al. to detect each parameter [26]. In order to measure both placental thickness and the retroplacental myometrium thickness, we positioned the probe so that the ultrasound beam was perpendicular to the uterine wall. The placental thickness was assessed at its thickest point. To measure the retroplacental myometrium, we enlarged the image to allow the accurate measurement of the hypoechoic muscle layer behind the placenta, obtaining the smallest myometrial thickness in the sagittal view. The retroplacental myometrial blood flow was assessed using color Doppler (CD) with a full bladder, with increased blood flow defined as a velocity of 20 cm/s or greater. In the sagittal plane, normal blood flow appeared as scattered, discontinuous signals in the uterine wall behind the placenta or as a narrow, uniform color strip representing a vessel. Enhanced blood flow, however, was characterized by thickened, winding vessels that appeared as overlapping, multi-colored structures or displayed a turbulent flow along the uterine wall. To study the length and vascularization of the cervix, the transvaginal approach was preferred. The shortened cervix was defined as a cervical length of less than 30 mm. The “riddled cervix” was defined when multiple cervical lakes or a shortened length were present.

## 4. Statistical Analysis

We used binary logistic regression analysis to calculate odds ratios (ORs) and 95% confidence intervals (95% CIs) to describe the associations between the ultrasound features and the histology of PAS and surgical complications. We performed a multivariate logistic regression analysis to select the most significant variables to predict the risk of PAS and surgical complications. Statistically significant sonographic features were selected and included in a final scoring system to calculate a total score of prediction of PAS and surgical complications. We used receiver operating characteristic (ROC) curves to calculate the thresholds for the total score that discriminated between PAS and no PAS. *p* < 0.05 was considered statistically significant. Comparison between women with and without PAS was carried out by an unpaired Student’s *t* test, and the total score was summarized by medians and quartiles and compared between the two groups by the Mann–Whitney U test. SAS v9.4 (SAS Institute, 2022 ©, Cary, NC, USA) was used for statistical analysis.

## 5. Results

### 5.1. Descriptive Analyses

#### 5.1.1. General Population Features

A total of 122 patients were initially selected. Among these, 20 patients were excluded because they had major systemic pathologies and did not meet the inclusion criteria. The study population comprised the remaining 102 pregnant patients who were treated at Hospital of Bari in the years 2018–2023. The mean maternal age at diagnosis was 34.2 years (SD 5.49). The mean gestational age at diagnosis was 24 weeks (SD 6). Of the cases, 3 were diagnosed in the first trimester (3%); 53 in the second trimester (53%); and 46 in the third trimester (46%). 13 patients underwent in vitro fertilization (13%). A history of cesarean section was present in 42 patients (42%). Of these patients, 30 had only had one previous CS (29%), while 12 had had more than one (12%). A total of 30 patients (29%) had a history of previous surgery on the uterus (14 had a previous myomectomy, 14%; 18 had uterine curettages, 18%).

#### 5.1.2. Sonographic Features

**Figure 1:**
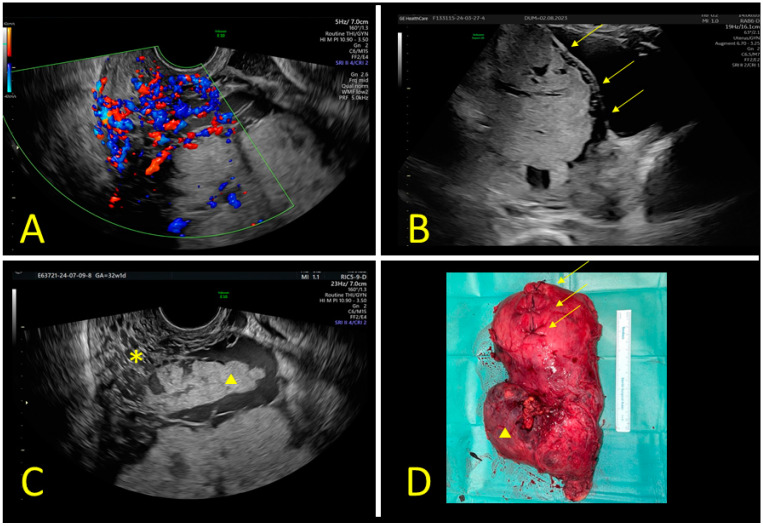
Sonographic findings in a case of placenta percreta. (**A**) “Riddled cervix” sign at 28 weeks of gestation. Color Doppler transvaginal scan of a highly vascularized (Color Score 3–4) cervix with multiple vascular lakes. Normal cervical length: 35 mm. (**B**) Same patient at 33 weeks of gestation: evidence of multiple white line interruptions (yellow arrows). (**C**) Vascular lacunae sign at 32 weeks. Para-sagittal right sonographic scan with evidence of a riddled cervix sign (*) and a right placental cotyledon (yellow triangle) surrounded by large peripheral vascular lacunae with bulging. Absent myometrial thickness. (**D**) Anatomical specimen of the uterus after cesarean section and subsequent hysterectomy at 34 weeks of gestation. Evidence of the longitudinal incision on the fundus (yellow arrows). Placenta previa percreta on the right isthmic side (yellow triangle) In this case, there was a riddled cervix sign, correlated with parametrial invasion.

The sonographic features of the population were as follows: anterior placenta, observed in 49 patients (48%), retroplacental space loss in 12 cases (12%), and 19 cases with thinning in the placental thickness (19%). Vascular lacunae were present in 35 patients (34%), and 12 (12%) exhibited white line interruption. The exophytic mass was a rare feature, only found in two patients (2%). We found intracervical lakes and/or a shortened cervix in 14 patients (14%). We highlighted the “riddled cervix” in three patients (3%). Retroplacental vascularization was found in 14 patients (14%), and 6 (6%) had bridging vessels. A pictorial of the “riddled cervix” sonographic findings is shown in Figure 1.

#### 5.1.3. Obstetrical and Delivery Characteristics

All patients with sonographic concern for placenta accreta underwent a cesarean section. The median gestational age at delivery was 36 weeks. In total, 83 patients underwent an elective cesarean section after at least 36 weeks of pregnancy (83%). Within this group, 10 patients, whose diagnosis was made after delivery on the histologic specimen, delivered at 38 (8%) or 39 (2%) weeks of pregnancy. A total of 19 patients had urgent delivery at a gestational age < 36 weeks (19%), and 89 patients had a transverse hysterotomy (89%), while 13 patients had a longitudinal hysterotomy (13%). The embolization of the hypogastric arteries was performed in eight patients (8%).

#### 5.1.4. Surgical Complications

In total, 41 patients had surgical complications: 14 patients underwent hysterectomy (14%); 3 had bladder lesions (3%); 2 had bowel lesions (2%); and 3 had major uterine atony (3%). A total of 32 patients (32%) had hemorrhagic complications (>1000 mL of blood loss); within this group, 12 needed hemostatic devices (Bakri Baloon©, Cook Medical LLC, Bloomington, Indiana, USA) (12%), and 25 patients needed at least a red blood cell unit transfusion (25%) Table 2.

### 5.2. PAS Score Assessment

The total scores had a median of 2, with an interquartile range of 1 to 3 (Table 3).

### 5.3. Logistic Regression Analysis

#### 5.3.1. Risk of PAS Disorder

The variable assignment method for binary logistic regression is shown in Table 4. The dependent variables were no PAS (n = 19) and PAS (n = 83). The independent variables were a history of cesarean section and a history of uterine surgery, anterior placenta, the loss of the retroplacental space, the thinning of the retroplacental myometrium, the presence of at least three placental lacunae, bladder white line interruption, the presence of exophytic mass, the presence of intracervical lakes or a shortened cervix, retroplacental myometrial blood flow, and the presence of bridging vessels. The Wald test for logistic regression showed that the model was correctly specified (*p* < 0.0001).

Features like a history of cesarean section, retroplacental space loss, myometrial thickness thinning, vascular lacunae, white line interruption, retroplacental vascularization, and bridge vessels show significantly high odds ratios, indicating a strong association with PAS, with *p*-values <0.0001 in most cases, suggesting strong statistical significance. The presence of intracervical lakes and a shortened cervix (OR 6.33, *p* = 0.003) were also significantly associated with PAS. Conversely, features like placental location, a history of uterine surgery, exophytic mass, and the “riddled cervix” sign show no significant association (*p* > 0.05). The overall score has an OR of 2.53, with a 95% CI of 1.65–3.87 and a *p*-value of <0.0001 (Wald test), indicating that the composite score significantly predicts PAS. The presence of retroplacental myometrial blood flow was the most important indicator of PAS, followed by reduced thickness in the retroplacental myometrium (<1 mm).

We performed a univariate logistic regression of the three most significant variables, which is shown in Table 5.

#### 5.3.2. Risk of PAS Complications

The variable assignment method for binary logistic regression is shown in Table 6. The dependent variables were no complications (n = 61) and complications (n = 41). The independent variables were a history of cesarean section and a history of uterine surgery, anterior placenta, the loss of the retroplacental space, the thinning of the retroplacental myometrium, the presence of at least three placental lacunae, bladder white line interruption, the presence of exophytic mass, the presence of cervical lakes or a shortened cervix, retroplacental myometrial blood flow, and the presence of bridging vessels (Table 6). The Wald test for logistic regression showed that the model was correctly specified (*p* < 0.0001).

Features like a history of cesarean section, the presence of at least three vascular lacunae, white line interruption and bridge vessels, the presence of cervical lakes or a shortened cervix show significantly high odds ratios, indicating a strong association with complications, with *p*-values < 0.0001 in most cases, suggesting strong statistical significance. Conversely, features like placental location, retroplacental space loss, retroplacental vascularization, the thinning of the retroplacental myometrium, a history of uterine surgery, and exophytic mass show no significant association (*p* > 0.05). The overall score has an OR of 1.89, with a 95% CI of 1.4–2.56 and a *p*-value of <0.0001 (Wald test), indicating that the composite score significantly predicts complications.

### 5.4. Multivariate Logistic Regression

#### 5.4.1. Multivariate Logistic Regression: Risk of PAS

We performed a multivariate logistic regression for the prediction of PAS, focusing on three key sonographic features: the thinning of the retroplacental myometrium, the presence of at least three vascular lacunae, and retroplacental vascularization (Table 7). The analysis demonstrates that the thinning of the retroplacental myometrium has the highest predictive value, with an OR of 32.77 (95% CI: 3.98–269.86, *p* = 0.001), followed by retroplacental vascularization, with an OR of 34.54 (95% CI: 2.58–462.43, *p* = 0.007), and vascular lacunae, which has an OR of 20.55 (95% CI: 1.23–342.50, *p* = 0.03). The diagnostic usefulness was categorized as absent for AUC ≤ 0.5, low for 0.5 < AUC ≤ 0.75, moderate for 0.75 < AUC ≤ 0.9, high for 0.9 < AUC < 0.97, and very high for AUC ≥ 0.97. The overall model performance, reflected by an area under the curve (AUC) of 0.98, indicates a very high predictive accuracy for PAS based on these features.

#### 5.4.2. Multivariate Logistic Regression: Risk of Complications

We performed a multivariate logistic regression for the prediction of complications, highlighting two features: vascular lacunae and white line interruption (Table 8). The odds ratio (OR) for vascular lacunae is 5.11, with a 95% confidence interval (CI) of 2.01–13.01, and a highly significant *p*-value of 0.0006, suggesting a strong association with complications. The second feature, white line interruption, has an OR of 5.66 (95% CI: 1.06–30.06, *p* = 0.04), indicating that it is also a significant predictor. The model’s overall performance is modest, with an area under the curve (AUC) of 0.72, reflecting a fair level of accuracy in predicting complications based on these two features.

## 6. Discussion

### 6.1. Main Results

The risk stratification of women affected by a PAS disorder is challenging [30,31]. Although advances in prenatal imaging have led to an increase in the detection rate of these anomalies, there is still limited evidence on how to identify cases at a higher risk of severe surgical morbidity [32,33,34]. In this study, we aimed to evaluate the experience of our center in diagnosing and managing PAS in a very high-risk population. The sonographic features used to assess the risk of PAS were those present in the main scoring systems [1,26,29]. The findings highlighted the clinical utility of prenatal ultrasound features in predicting PAS severity and surgical complications [35,36,37]. We identified significant associations between specific sonographic markers (retroplacental myometrial thinning, multiple vascular lacunae, and retroplacental vascularization) and poor maternal outcomes, confirming the diagnostic value of these features. This is in line with findings from Zhang et al., who emphasized the role of placental lacunae and myometrial thinning as critical markers in PAS diagnosis [29]. Similarly, our results showed that myometrial thinning (<1 mm) and the presence of retroplacental vascularization were the strongest predictors of PAS and surgical complications, with odds ratios indicating a significant association with maternal morbidity. These findings support the work by Rac et al., who developed the Placenta Accreta Index (PAI), highlighting the diagnostic importance of such sonographic parameters [26]. Only a few studies have attempted to explore the feasibility and diagnostic performance of an ultrasound-based scoring system in assessing the presence and severity of PAS disorders [27,38]. Tovbin et al. [39] reported that a scoring system including the number of placental lacunae and the presence of bladder wall interruption had a high diagnostic performance for PAS, with an area under the receiver–operating characteristics curve of 0.94 (95% CI, 0.86–1.0), while, in the study by Rac et al. [26], the combination of the smallest sagittal myometrial thickness and the presence of lacunae and bridging vessels, in addition to the number of previous cesarean deliveries and placental location, yielded an area under the curve of 0.87 (95% CI, 0.80–0.95).

### 6.2. Diagnostic Accuracy of PAS Signs

The high predictive accuracy of our scoring system (AUC 0.98) aligns with previous research suggesting that ultrasound-based scoring systems can effectively stratify PAS risk [40]. Cali et al. introduced a PAS ultrasound staging system that similarly demonstrated significant correlations between ultrasound features and surgical outcomes [1]. However, unlike some of these earlier studies, our study focused specifically on a very high-risk population, where the incidence of PAS was expected to be higher, potentially increasing the predictive value of certain features and underestimating some others, such as the method of conception (spontaneous vs. in vitro fertilization), which, in our study, was evident in placenta previa but not significantly related to PAS.

One important finding was the correlation between cervical or parametrial involvement and the presence of the “riddled cervix”, a novel ultrasound marker identified in 3% of our study population [41]. This rare sign indicates significant deep cervical and parametrial involvement and has shown promise as a predictor of complications, highlighting the value of detailed cervical assessment in patients with suspected PAS. However, due to the rarity of this sign and the limited sample size, the statistical analysis did not reveal any significant association with PAS or related complications, despite the sign’s potential validity. The topography of placental invasion has recently been proposed as a reliable predictor of surgical morbidity in women affected by a PAS disorder [42]. Invasion in the inferior third of the lower uterine segment, bladder, and parametria carries a high risk of surgical morbidity, while upper invasions are commonly associated with a more favorable clinical outcome and a relatively easier vascular control surgery [43]. The assessment of the topography of invasion has been reported only on MRI, and it is still unclear whether such staging can be reproduced on ultrasound [44,45].

### 6.3. Study Strength

The large sample size and the longitudinal assessment of the included women (from the time of referral to our center to the delivery) represent the major strengths of this study. Furthermore, we evaluated the correlation of the proposed staging system for PAS disorders not only with surgical outcome but also with the depth of placental invasion and the FIGO grading system. Finally, all cases affected by PAS were managed by the same multidisciplinary team, thus reducing bias related to the operator’s experience.

### 6.4. Study Limitations

Some limitations must be acknowledged. First, this study was retrospective, which may introduce selection bias. Although our population consisted of very high-risk women, the relatively small sample size limits the generalizability of our findings. Additionally, while the predictive power of our scoring system was high, it may not perform equally well in lower-risk populations. Finally, the histopathologic confirmation of PAS was not uniformly available for all patients, which could impact the accuracy of PAS diagnosis in some cases. Despite the promising diagnostic performance of the ultrasound features, challenges remain. Notably, the interobserver variability in ultrasound interpretation continues to be a limitation, as previously identified by Zhang et al. [29,46]. Moreover, the absence of a standardized diagnostic protocol across institutions complicates the generalizability of our findings [47,48]. Further research is needed to validate the “riddled cervix” sign in broader populations and to refine PAS scoring systems, particularly in cases where parametrial involvement is suspected [41].

## 7. Conclusions

Our study confirms the utility of specific ultrasound features—particularly myometrial thinning, retroplacental vascularization, and the “riddled cervix” sign—in predicting PAS and its associated surgical complications. The high diagnostic accuracy of these markers, especially in high-risk populations, supports their inclusion in standardized PAS diagnostic protocols. Future research should focus on validating these findings across broader populations and refining scoring systems to improve prenatal risk stratification and the management of PAS.

## Figures and Tables

**Table 1:** Proposed scoring system.

Risk Factors and Sonographic Features	Score
Previous cesarean section	1
Other surgery on uterus	1
Placental location (anterior)	1
Presence of >3 placental lacunae	1
Absence of retroplacental space	1
Bladder line interruption	1
Presence of exophytic mass	1
Thickness of retroplacental myometrium (<1 mm)	1
Retroplacental myometrial blood flow	1
Presence of bridging vessels	1
Presence of intracervical lakes/shortened cervix	1

**Table 2:** Observed complications and frequency.

Complications	
Hysterectomy, N (%)	14 (14%)
Hematic loss of more than 1000 mL, N (%)	32 (32%)
Bladder lesion, N (%)	3 (3%)
Bowel lesion, N (%)	2 (2%)
Major uterine atony, N (%)	3 (3%)
At least one red blood cell unit transfusion, N (%)	25 (25%)
Hemostatic devices, N (%)	12 (12%)

**Table 3:** Population’s observed features and their frequency.

Features	
Maternal age, mean (SD)	34.2 (5.4%)
History of cesarean section, N (%)	42 (42%)
History of uterine surgery, N (%)	30 (29%)
Anterior placenta, N (%)	49 (48%)
Retroplacental space loss, N (%)	12 (12%)
Myometrial thickness thinning, N (%)	19 (19%)
Vascular lacunae, N (%)	35 (34%)
White line interruption, N (%)	12 (12%)
Exophytic mass, N (%)	2 (2%)
Intracervical lakes/shortened cervix, N (%)	14 (14%)
Riddled cervix, N (%) Retroplacental vascularization, N (%)	3 (3%) 14 (14%)
Bridging vessels, N (%)	6 (6%)
Total score, median (Q1–Q3)	2 (1–3)

**Table 4:** Univariate logistic regression analysis results for PAS risk.

Feature and Score	OR	95% CI	*p*-Value
History of cesarean section	7.78	2.35–25.69	<0.001
History of uterine surgery	0.83	0.27–2.55	0.74
Anterior placenta	2.83	0.98–8.17	0.055
Retroplacental space loss	44.99	8.50–238.35	<0.0001
Thinning of the retroplacental myometrium	142.22	26.30–769.15	<0.0001
At least three vascular lacunae	30.69	6.48–145.40	<0.0001
White line interruption	14.36	3.70–55.73	0.0001
Exophytic mass	4.56	0.27–76.30	0.29
Intracervical lakes or shortened cervix	6.33	1.88–21.27	0.003
Retroplacental vascularization	177.67	19.76–∞	<0.0001
Bridging vessels	29.29	3.18–269.79	0.003
Score	2.53	1.65–3.87	<0.0001

**Table 5:** Univariate logistic regression analysis results for PAS.

Feature	OR	95% CI	Graph Lower 95% CI	95% CI	Graph Upper 95% CI		*p*-Value
Myometrial thickness thinning	32.77	3.98	28.79	269.86	237.09	0.5	0.001
Vascular lacunae	20.55	1.23	19.32	342.5	321.95	1.5	0.03
Retroplacental vascularization	34.54	2.58	31.96	462.43	427.89	2.5	0.007

**Table 6:** Univariate logistic regression analysis results for complications.

Feature and Score	OR	95% CI	*p*-Value
History of cesarean section	2.82	1.24–6.42	0.01
History of uterine surgery	1.20	0.51–2.85	0.68
Anterior placenta	1.72	0.77–3.82	0.18
Retroplacental space loss	Undefined		
Thinning of the retroplacental myometrium	Undefined		
At least three vascular lacunae	6.42	2.61–15.80	<0.0001
White line interruption	9.52	1.96–46.16	0.005
Exophytic mass	1.5	0.09–24.68	0.78
Cervical lakes or a shortened cervix	4.60	1.33–15.87	0.02
Retroplacental vascularization	Undefined		
Bridging vessels	8.33	0.94–74.19	0.06
Score	1.89	1.4–2.56	<0.0001

**Table 7:** Multivariate logistic regression analysis for PAS.

Feature	OR	95% CI	*p*-Value
Myometrial thickness thinning	32.77	3.98–269.86	0.001
Vascular lacunae	20.55	1.23–342.50	0.03
Retroplacental vascularization	34.54	2.58–462.43	0.007
AUC: 0.98			

**Table 8:** Multivariate logistic regression analysis for complications.

Feature	OR	95% CI	*p*-Value
Vascular lacunae	5.11	2.01–13.01	0.0006
White line interruption	5.66	1.06–30.06	0.04
AUC: 0.72			

## Data Availability

All data are reported in the text.
